# Network Dynamics of a Financial Ecosystem

**DOI:** 10.1038/s41598-020-61346-y

**Published:** 2020-03-12

**Authors:** Shahar Somin, Yaniv Altshuler, Goren Gordon, Alex ’Sandy’ Pentland, Erez Shmueli

**Affiliations:** 1 Industrial Engineering Department, Tel Aviv University, Tel Aviv, Israel; 2 MIT Media Lab, Cambridge, MA USA; 3 Endor Ltd., Tel Aviv, Israel

**Keywords:** Applied mathematics, Complex networks

## Abstract

Global financial crises have led to the understanding that classical econometric models are limited in comprehending financial markets in extreme conditions, partially since they disregarded complex interactions within the system. Consequently, in recent years research efforts have been directed towards modeling the structure and dynamics of the underlying networks of financial ecosystems. However, difficulties in acquiring fine-grained empirical financial data, due to regulatory limitations, intellectual property and privacy control, still hinder the application of network analysis to financial markets. In this paper we study the trading of cryptocurrency tokens on top of the Ethereum Blockchain, which is the largest publicly available financial data source that has a granularity of individual trades and users, and which provides a rare opportunity to analyze and model financial behavior in an evolving market from its inception. This quickly developing economy is comprised of tens of thousands of different financial assets with an aggregated valuation of more than 500 Billion USD and typical daily volume of 30 Billion USD, and manifests highly volatile dynamics when viewed using classic market measures. However, by applying network theory methods we demonstrate clear structural properties and converging dynamics, indicating that this ecosystem functions as a single coherent financial market. These results suggest that a better understanding of traditional markets could become possible through the analysis of fine-grained, abundant and publicly available data of cryptomarkets.

## Introduction

Classic econometric models were demonstrated to be limited in explaining crowd phenomena such as economic cycles and crashes, partially since they do not explicitly account for the complex interactions within the economic system. Aiming to achieve a better understanding of such macroscopic effects, recent years have witnessed a rising interest in the application of network theory for analyzing the underlying networks of economic systems^1–10^. However the general lack of large-scale, fine-grained empirical financial data, due to regulatory limitations, intellectual property restrictions and privacy regulation, still hinders the full exploitation of such techniques for analyzing complex financial markets.

In this paper we study the complete financial activity of millions of participants in an economy comprising of thousands of different financial assets over a period of 2.5 years. To this end, we use the publicly available Ethereum blockchain transactional data^11^. This data encompasses the complete trading activity of 28 Million users trading over 11 thousand assets^12–14^ of aggregated market valuation peeking at 500 Billion USD with a daily trading volume of over 30B USD^15^. In contrast to traditional financial markets, all assets’ transactions over the Ethereum Blockchain are permanently recorded and publicly accessible. As a result, the Ethereum Blockchain constitutes the world’s largest publicly available financial dataset at an individual trader granularity. This dataset grants us for the first time the opportunity to analyze and model a dynamic large scale financial ecosystem, from its inception throughout its evolution.

In this work we first demonstrate that the Ethereum financial ecosystem is highly diverse, as manifested by the varied designations, wide-ranging lifespans and popularity levels of its traded assets. We then show that this market also presents highly volatile dynamics when viewed using classical economic measures. Notwithstanding the above-mentioned multi-faceted and erratic nature, we substantiate that this complex ecosystem functions as a single market for thousands of types of transactions, when analyzed using network oriented methodologies. The latter is established in two complementing aspects — both by applying a static analysis on its underlying networks of interactions, affirming they adhere to a robust, well-defined structure, and by applying a dynamical analysis, ascertaining it has a characteristic network evolution and convergent dynamics.

More specifically, our static analysis examines the underlying networks of trading interactions by constructing a stroboscopic-like snapshots of the emerging network. We demonstrate that the vast majority of these static networks follow a power-law-like degree distribution, similarly to many real-world networks^16–23^, ascertaining their characteristic structure.

We then analyze the dynamics of the evolution of this ecosystem’s underlying network. We start by analyzing *γ*, the exponent of the degree distribution, assuming it follows a power-law distribution, along time. This analysis reveals a remarkably stable dynamics during the last 1.5 years of activity. Unfortunately, a *γ*-based analysis of the first year of financial activity is mostly implausible, mainly due to the small networks’ sizes during this time, impeding statistically significant estimates of its value. Given that this year constitutes the establishment stage of the Ethereum economy, analyzing its dynamics during this initial period is of great significance.

We therefore propose a new measure which is based on the network’s degree distribution, focusing on its two extrema points—the max-connected node and the 1-connected nodes. The new measure is also applicable to small sized networks, and as such, it can be used to analyze the dynamics of this extremely complex ecosystem throughout the entire examined period, including its establishment stage. We demonstrate that the dynamics of the Ethereum economy, as captured by this new measure, can be modeled using an under-damped harmonic oscillator, substantiating the equilibration of this economy over time.

These global structural and dynamical patterns indicate that the Ethereum ecosystem acts as a single financial market of buyers and sellers, although being comprised of thousands of different, seemingly unrelated or correlated assets. Moreover, these patterns advocate a strong similarity between the Ethereum trading ecosystem and traditional financial markets, which are also known to be characterized by power-law degree distributions^8,24–27^ and oscillatory dynamics^28,29^. This similarity suggests that a better understanding of traditional financial markets could be achieved through the modeling of the Ethereum economy, where data is ample and fine-grained.

## Results

Contrary to the Bitcoin Blockchain^30–32^ which supports the trading of only a single asset, the Ethereum Blockchain is comprised of thousands of diverse assets, where new traded goods are constantly emerging and disappearing, forming a highly dynamic and heterogeneous economy. We analyzed 180 million transactions over 2.5 years, between April 2016 and January 2019, encompassing the trading of 11940 assets by 28 million different wallets (representing the individual trading entities). Our analysis was restricted to *active* assets, each participating in at least 100 trades throughout the analyzed timespan.

### Emerging structure

We first demonstrate how heterogeneous the Ethereum economy actually is. The diversity of this financial ecosystem can be observed first and foremost through the variety of traded assets and their functionalities, ranging from trading in gold (CrytoGold), to Blockchain-related predictions (Bancor^33^), to decentralized storage (Filecoin^34^), and scalable smart contracts platform (EOS^35^).

**Figure 1:**
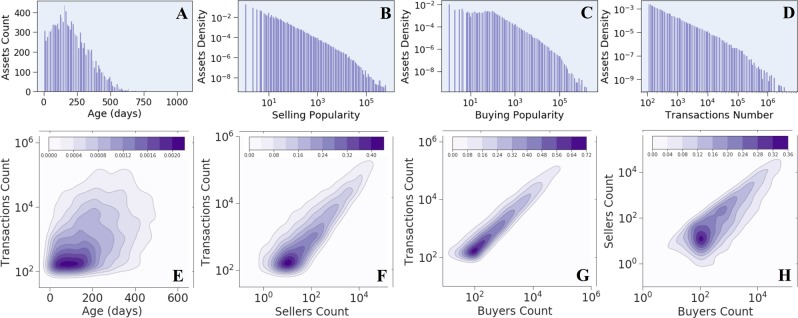
Marginal (upper panels) and joint (lower panels) distributions of various aspects of the traded assets, reflecting the diversity of the Ethereum economy. Panel A depicts assets’ age distribution, panels B–D represent the distribution of various popularity measures of assets (selling, buying and trading volume, respectively). Panel E–G present the joint distribution of the trading volume against assets age, selling and buying popularities correspondingly. Panel H presents the joint distribution of selling and buying popularities. All joint distributions are estimated using a bivariate KDE (Kernel Density Estimation). The units in the color-bar represent the estimations of the corresponding probability density functions by the bivariate KDE.

Apart from their functionalities, the traded goods also vary in their characteristics. Panel A in Fig. 1 depicts assets’ age distribution, where the age of an asset is calculated as the number of days passed between the first and last time it was traded. We further evaluate the popularity levels for the traded assets. Specifically, we define the selling popularity of an asset as the number of unique traders who ever sold that asset (and analogically define the buying popularity of an asset). Panel B and C in Fig. 1 depict the distributions of selling and buying popularities among assets. Panel D depicts the distribution of yet another notion of assets’ popularity, the trading volume. We note that all three popularity measures present heavy-tailed distributions. These characteristics reflect the diverse nature of this economy, as it comprises the trading of very old assets along with recently minted ones, and popular assets along with barely traded ones.

In order to further substantiate the inherent diversity of this financial ecosystem, we analyze the joint distributions of assets’ characteristics. Panel E in Fig. 1 depicts the joint distribution of assets’ age and trading volume. It implies that in this ecosystem popularity does not necessarily accumulate with time (age), a rather surprising observation since one might expect that old assets would accumulate the highest trading volume during their long lifespan. Furthermore, panels F and G depict the joint distribution of trading volume and selling and buying popularities, respectively. Panel H manifests the joint distribution of selling and buying popularities of assets. We see that the joint distributions of these three different popularity measures depict significant spread, despite the intuitive expectation of high correlation between them.

We conclude that the traded assets in this economy have diverse types and functionalities, varied ages, trading volume and popularity, yet reside together in a single multi-faceted ecosystem. Our first goal in this paper will be to show that despite the inherent heterogeneity of this economy, it has a robust underlying structure. To that end, we analyze the Ethereum economy using a network theory perspective. We define the weekly transactions graph of the Ethereum economy at time *t* as follows:

#### Definition 1.

The weekly transactions graph for a given day *t*, *G*_*t*_(*V*_*t*_, *E*_*t*_) is the directed graph constructed from all trading transactions over any traded asset, made during the time period [*t* − 7, *t*). The set of vertices *V*_*t*_ consists of all wallets trading during that period:

1 $${V}_{t}:=\,\{v\parallel \,{\rm{w}}{\rm{a}}{\rm{l}}{\rm{l}}{\rm{e}}{\rm{t}}\,v\,{\rm{b}}{\rm{o}}{\rm{u}}{\rm{g}}{\rm{h}}{\rm{t}}\,{\rm{o}}{\rm{r}}\,{\rm{s}}{\rm{o}}{\rm{l}}{\rm{d}}\,{\rm{a}}{\rm{n}}{\rm{y}}\,{\rm{a}}{\rm{s}}{\rm{s}}{\rm{e}}{\rm{t}}\,{\rm{d}}{\rm{u}}{\rm{r}}{\rm{i}}{\rm{n}}{\rm{g}}\,[t-7,t)\}$$

and the set of edges *E*_*t*_ ⊆ *V*_*t*_ × *V*_*t*_ is defined as:

2 $${E}_{t}:=\,\{(u,v)\parallel \,{\rm{w}}{\rm{a}}{\rm{l}}{\rm{l}}{\rm{e}}{\rm{t}}\,u\,{\rm{s}}{\rm{o}}{\rm{l}}{\rm{d}}\,{\rm{t}}{\rm{o}}\,{\rm{w}}{\rm{a}}{\rm{l}}{\rm{l}}{\rm{e}}{\rm{t}}\,v\,{\rm{a}}{\rm{n}}{\rm{y}}\,{\rm{a}}{\rm{s}}{\rm{s}}{\rm{e}}{\rm{t}}\,{\rm{d}}{\rm{u}}{\rm{r}}{\rm{i}}{\rm{n}}{\rm{g}}\,\,[t-7,t)\}$$

Over the examined period of 2.5 years, we construct 1000 such weekly transactions graphs, using daily rolling windows, each containing one week of transactional data.

**Figure 2:**
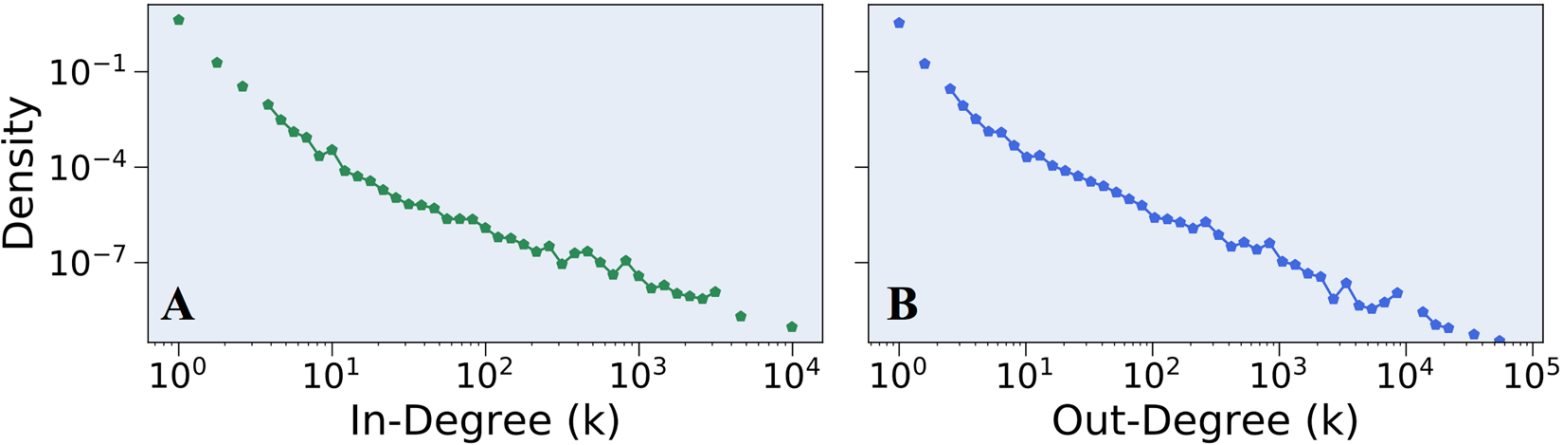
Incoming and outgoing degree distributions of a weekly transactions graph, generated for the week of February 1^st^, 2018. Both degree distributions are plotted on a log-log scale, with logarithmic binning, normalized by bin-width. Both appear to follow a heavy-tailed distribution.

Numerous previous works have shown that degree distributions of complex systems and economic systems in particular are often heavy-tailed. Fig. 2 depicts the incoming and outgoing degree distributions of an arbitrary weekly transactions graph, suggesting that the degree distribution of weekly snapshots of the Ethereum economy is heavy tailed, coinciding with such expectations. Consequently, we calculate both incoming and outgoing degree distributions for each of the 1000 weekly transactions graphs. We then apply prevalent statistical analysis methods^36^ to evaluate which of four potential heavy-tailed models best fits the examined degree distributions.

**Figure 3:**
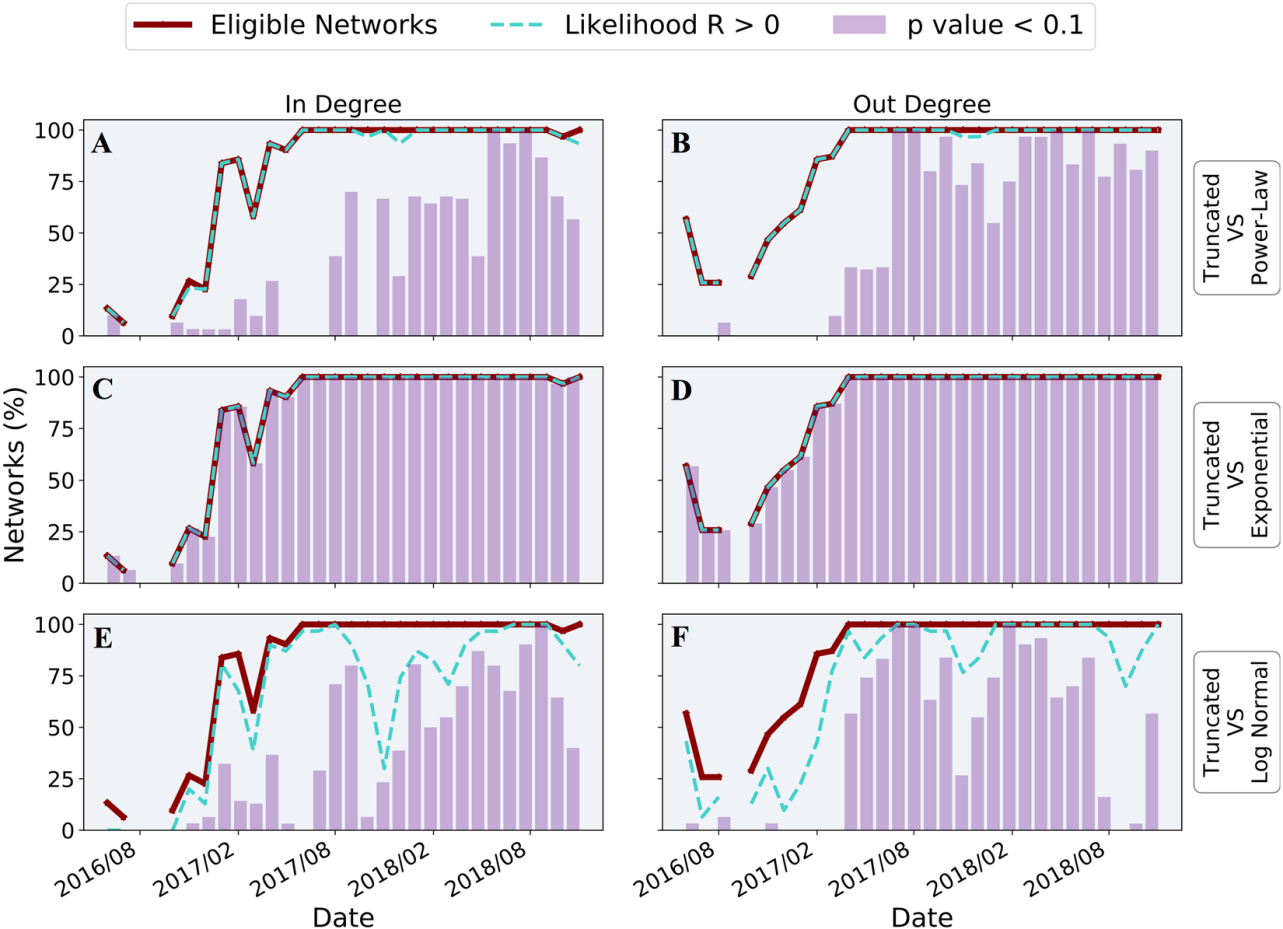
Networks structure analysis. Presenting the analysis of incoming (left panels) and outgoing degrees (right panels) of 1000 weekly networks. The solid line specifies the monthly percentage of weekly networks eligible for statistically significant LLR calculations (having over 50 different degrees, same for all panels). The dotted line presents the monthly percentage of networks having a positive log-likelihood ratio when comparing the truncated power-law model to power-law (panel A, B), to exponential (panels C, D) and to log-normal (panels E, F) hypotheses. The bars stand for the monthly percentage of networks whose LLR calculation is statistically significant, obtaining p-value < 0.1. The structures of the examined weekly networks present high agreement with the truncated power-law model.

In order to guarantee reliable parameters’ estimates, we consider only networks that have at least 50 unique node degrees^36^. The solid line in Fig. 3 (same for all panels) shows the monthly percentage of such eligible networks. Out of the 2000 (in and out) examined networks, 76% were found to be eligible. In particular, starting from April 2017, alongside with a significant increase in networks’ sizes (see Supplementary Fig. S1 for nodes’ temporal dynamics), the vast majority of networks reach the designated threshold of unique incoming and outgoing degrees.

For all eligible networks, we perform a *goodness of fit* test calculating the Log-Likelihood Ratio (LLR) of the different models and the corresponding p-values. Panels A and B in Fig. 3 present the comparison of the truncated power-law to the power-law model for incoming and outgoing degree, respectively, along time. Specifically, the dashed line stands for the monthly percentage of networks obtaining a positive LLR, while the bars signify the percentage of networks for which this test achieved p-value < 0.1. The results suggest that the truncated power-law model better fits the majority of networks compared to the power-law model, due to the positive LLR obtained by 99% of all eligible networks, with 48% (63%) of the networks in the in (out)-degree case present statistically significant results.

This inclination of the degree distributions towards the truncated power-law model led us to continue and validate it against other heavy-tailed distributions as well. Panels C and D in Fig. 3 present the LLR of truncated power-law and the exponential model, alongside the corresponding p-values, demonstrating a clear dominance of the truncated power-law model, with 100% of the eligible networks obtaining positive LLR with p-value < 0.1, for both in and out degrees. Panels E and F in Fig. 3 demonstrate the LLR of truncated power-law and the log normal model accompanied by the corresponding p-values for both in and out degree distributions along time. Once again we observe a strong inclination towards the truncated power-law model with a total of 59% (61%) of the positive LLR networks for the in (out)-degree case presenting statistically significant outcome.

We conclude that starting from the second year of data, alongside with meeting the threshold conditions for significant statistical analysis, the vast majority of eligible networks presents a good agreement with the truncated power-law model, which is favored over alternative long-tailed distributions, with high statistical significance. Our findings indicate that despite the high diversity apparent by the Ethereum economy, it manifests a robust underlying structure.

### Converging dynamics

**Figure 4:**
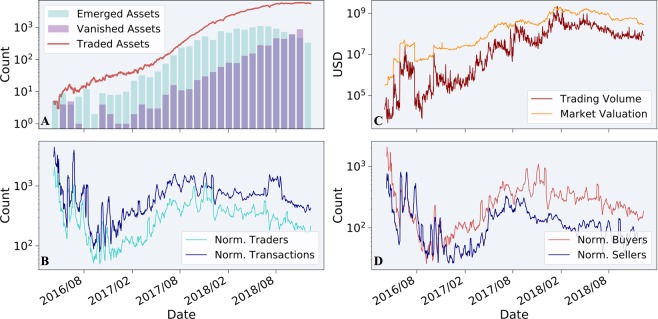
Dynamics of traditional measures of the Ethereum economy. Solid line in panel A depicts the number of traded assets over time. Bars in panel A indicate the number of monthly emerged and vanished assets. Specifically, number of emerged assets for a given month stands for the number of assets having their first trade during that month (and analogically for monthly vanished assets). Panel B presents the number of traders (wallets) and number of transactions over time. Panel C presents top-5 assets’ (sorted by market valuation) trading volume and their corresponding averaged market valuation. Panel D depicts the dynamics of weekly number of buyers and sellers. Numbers of weekly traders, transactions, buyers and sellers are all normalized by the number of distinct assets traded during the corresponding week. All measures are presented at log-scale.

Aiming to explore and comprehend the dynamics of this diverse financial ecosystem over time, we start by employing several classical economic measures. Fig. 4 depicts the dynamics of several intrinsic properties of the ecosystem: number of total traded assets alongside with the number of newly emerged assets and vanished ones (Panel A), normalized number of traders (wallets) and transactions (Panel B), top-5 assets’ (sorted by market valuation) trading volume and their corresponding market valuation (Panel C) and the normalized number of buyers and sellers (Panel D). These properties are inspected over daily rolling windows, each containing one week of transactional data, over the examined period of 2.5 years. As can be seen in panel A, the number of traded assets increases over time, resulting from the rising interest in Blockchain and crypto-economy throughout the examined timespan. Bars in panel A present the dynamic composition of the ecosystem, as new assets constantly emerge and others vanish along time. The trading volume, market cap and the normalized amounts of traders, transactions, buyers and sellers (panels B-D) manifest high volatility throughout the entire period.

**Figure 5:**
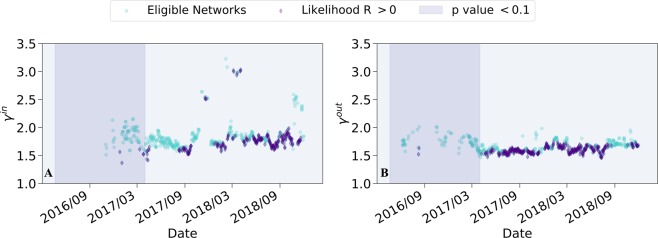
Dynamics of *γ*^*in*^ and *γ*^*out*^, restricted to networks with positive likelihood ratio and p-value < 0.1 when comparing the truncated power-law model to power-law, exponential and log-normal models. Both in and out *γ* parameters are stable along time. Statistically significant network dynamics during the first year of data (depicted by a darker patch, left side of each plot) are lacking.

Next, we show that despite the apparent volatile dynamics of this economy, applying a network theory prism reveals clear converging dynamics. We start by analyzing the number of nodes and edges in the weekly transactions graphs, over time (see Supplementary Fig. S1). We observe a rapid growth in the number of nodes and edges, starting from around April 2017. We continue by examining the weekly transactions graphs’ degree distributions over time. Specifically, we analyze their *γ* values — the exponent of the degree distribution, assuming it follows a truncated power-law. We apply a Maximum-Likelihood Estimator^36^ in order to evaluate *γ* for all corresponding truncated power-law distributions. Panels A and B in Fig. 5 depict the dynamics of *γ*^*in*^ and *γ*^*out*^ respectively. We restricted our analysis merely to networks that passed the *goodness of fit* tests (compared with power-law, exponential and log-normal distributions) and indicated the networks that obtained p-value < 0.1 in all three tests. As can be seen from the figure, this analysis depicts stable dynamics of both in and out *γ* parameters.

Unfortunately, the analysis above seems to provide little insight regarding the dynamics of the economy during its first year of activity. More specifically, until April 2017 (depicted by darker patch in Fig. 5), a significant amount of the networks is not eligible for *γ* calculation, and those which are, often present statistically insignificant results during this stage. This is most probably due to the relatively small size of networks during this period (see Panel A in Supplementary Fig. S1). Nevertheless, analyzing the networks’ dynamics during this initial period is of great significance since it constitutes the establishment stage of this economy.

We therefore examine a new aspect of the degree distribution, focusing on its *extrema points*, namely the number of 1-connected nodes (i.e number of traders who formed merely one transaction) and the degree of the max-connected node. Specifically, for incoming degree we denote by:

3 $$C{N}_{in}^{1}=\{v\in {V}_{t}\,||\,in{\rm{\_}}deg(v)=1\},\,\,C{N}_{in}^{max}=\mathop{{\rm{a}}{\rm{r}}{\rm{g}}{\rm{m}}{\rm{a}}{\rm{x}}}\limits_{v\in {V}_{t}}\,in{\rm{\_}}deg(v)$$

the set of 1-in-connected nodes and the max-in-connected node, respectively. Similarly, for the outgoing degree distribution, its *extrema points* are denoted by:

4 $$C{N}_{out}^{1}=\{v\in {V}_{t}\,||\,out{\rm{\_}}deg(v)=1\},\,\,C{N}_{out}^{max}=\mathop{{\rm{a}}{\rm{r}}{\rm{g}}{\rm{m}}{\rm{a}}{\rm{x}}}\limits_{v\in {V}_{t}}\,out{\rm{\_}}deg(v)$$

standing for the set of 1-out-connected nodes and the max-out-connected node. We then calculate the ratio between the *extrema points* of each distribution, formally defined by:

#### Definition 2.

Let *G*_*t*_(*V*_*t*_, *E*_*t*_) be a weekly transactions graph, the In-Degree Ratio of *G*_*t*_ is defined as follows:

5 $${R}_{in}({G}_{t})=\frac{{\rm{\log }}\,(| C{N}_{in}^{1}| )}{{\rm{\log }}\,(in\_deg(C{N}_{in}^{max}))}$$

Similarly, the Out-Degree Ratio of a network is defined as:

6 $${R}_{out}({G}_{t})=\frac{{\rm{\log }}\,(| C{N}_{out}^{1}| )}{{\rm{\log }}\,(out\_deg(C{N}_{out}^{max}))}$$

Note that the ratios *R*_*in*_ and *R*_*out*_ can be viewed as approximations of *γ*^*in*^ and *γ*^*out*^. In particular, given a network *G*_*t*_, with a degree-distribution s.t it’s *extrema points* are $$in\_deg(C{N}_{in}^{max})$$ and $$| C{N}_{in}^{1}| $$, had there been a *pure* power-law model to fit the in-degree distribution of *G*_*t*_ (and specifically its extrema points), it would have satisfied *γ*^*in*^ = *R*_*in*_. This is a straightforward derivation from the linear form of the power-law degree distribution: $${\rm{\log }}\,p(x)=\gamma \cdot logx+c$$. Unlike the *γ* estimation procedure, which is guaranteed to be unbiased only for large sample sizes^36^, *R*_*in*_ and *R*_*out*_ can be calculated even on small networks, such as the ones apparent during the initial period of the economy.

**Figure 6:**
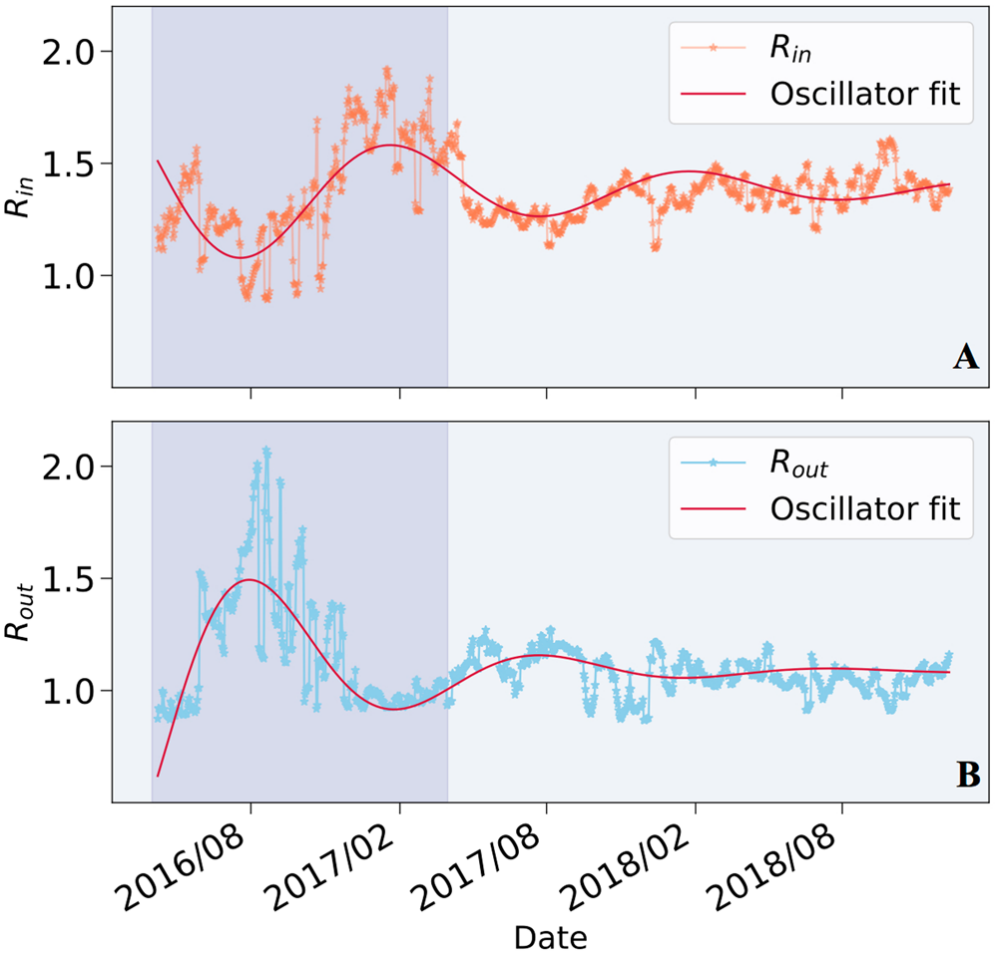
Dynamics of In-Degree (*R*_*in*_) and Out-Degree (*R*_*out*_) Ratios presented in panels A and B respectively. Both parameters are fitted by an Under-Damped Harmonic Oscillator, ascertaining the consolidation process undergone by the Ethereum economy.

Panels A, B in Fig. 6 present the dynamics of the ratios *R*_*in*_ and *R*_*out*_ along time. As can be seen from the figure, the dynamics of *R*_*in*_ and *R*_*out*_ are characterized by anti-phased oscillations that converge over time. Consequently, we model their dynamics over time *t* using an Under-Damped Harmonic Oscillator:

7 $$osc(t)=A\cdot {e}^{-{\omega }_{0}\zeta \cdot t}\cdot \sin ({\omega }_{0}\sqrt{1-{\zeta }^{2}}t+\varphi )+{R}^{\infty }$$

where *A* represents the maximal amplitude of the oscillation, *ω*_0_ is the resonant frequency of the system, *ζ* stands for the damping ratio, *φ* for the phase of the oscillation and *R*^*∞*^ for the equilibrium state. All parameters’ values of both fitted oscillator models are elaborated in Supplementary Table S1 and the residuals plots of both fits are displayed in Supplementary Fig. S2.

We chose to model these ratios’ dynamics using an under-damped harmonic oscillator for two reasons. First, it is able to capture various evident phenomena, ranging from oscillations, to decay and stabilization. Second, the interpretable nature of its parameters is helpful for extracting insights on the dynamics of this ecosystem. Specifically, they quantify and ascertain the anti-phased oscillations of *R*_*out*_ and *R*_*in*_, their different damping ratios, and correspondingly their different equilibrium states. A thorough discussion of these insights and hypotheses concerning their causes are presented in the Supplementary Material (Supplementary Figs. S4 and S5).

In order to verify that these under-damped oscillations are not a mere side effect of the network’s size, we examined the extent to which *R*_*out*_ and *R*_*in*_ are influenced by the number of nodes *N*. We establish that while *N* seems to influence the damping rate, it does not govern the oscillatory nature itself. The full analysis of the influence of *N* on the under-damped oscillatory dynamics of *R*_*out*_ and *R*_*in*_ is presented in Supplementary Fig. S3.

In essence, the oscillatory patterns exhibited by *R*_*out*_ and *R*_*in*_ substantiate our hypothesis that the Ethereum economy functions as a single market of buyers and sellers. These patterns also resonate well with known oscillatory dynamics of traditional economies, affirming remarkable resemblance between the Ethereum economy and traditional markets. Moreover, the dynamics of *R*_*out*_ and *R*_*in*_, as well as of *γ*-s, present significant stabilization after April 2017. This ascertains the convergence process undergone by the Ethereum financial system during the course of the examined 2.5 years, despite the otherwise volatile dynamics evident by traditional economic measures.

We conclude by presenting a quantitative analysis of the stability of *R*_*in*_ and *R*_*out*_ over time in comparison to some of the previously examined economic measures. Specifically, we analyze the Coefficient of Variation (CV) of all these parameters over time, where CV is defined as follows:

8 $$CV(x):=\frac{std(x)}{mean(x)}$$

**Figure 7:**
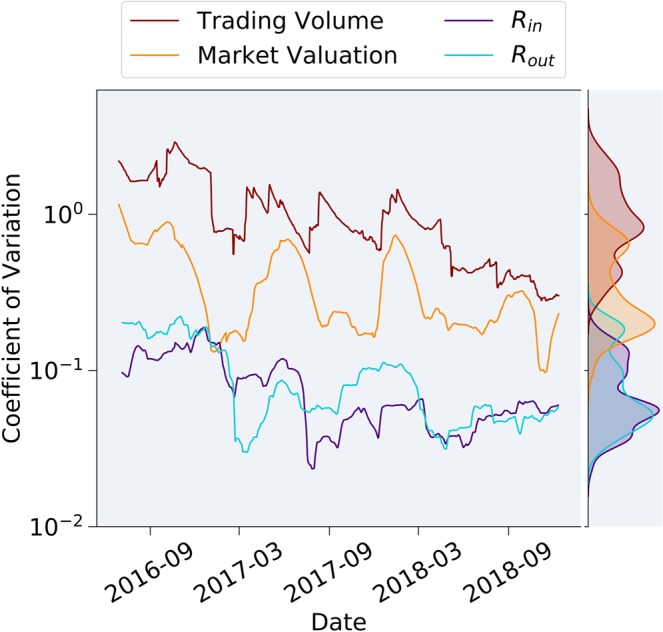
Coefficient of variation dynamics, comparing volatility over time of Out-Degree Ratio (*R*_*out*_) and In-Degree Ratio (*R*_*in*_) to traditional economic measures, such as the top-5 assets’ averaged market valuation and their average trading volume. Although both traditional and network-oriented measures present decreasing volatility along time, the higher coefficients of variation for the traditional parameters, attest to a less stable representation using traditional market indicators.

Fig. 7 displays the Coefficient of Variation of In-Degree and Out-Degree Ratios compared to the CV of the trading volume and market capitalization. It is evident that the variance of traditional economic measures is an order of magnitude higher than the CV of *R*_*in*_ and *R*_*out*_ throughout the entire examined timespan. This substantiates and quantifies our hypothesis that network oriented methodology enables a more stable representation of the economy compared to traditional market indicators.

## Discussion

In this study we analyzed the Ethereum financial ecosystem — the world’s largest publicly available fine-grained financial dataset. To the best of our knowledge this study is the first to analyze a complete financial activity of thousands of unrelated traded assets carried out by millions of traders over a long period of time. In particular, we presented evidence suggesting that the Ethereum ecosystem, despite being highly diverse in its assets’ composition, popularity, functionality, lifespan and trading volumes, still follows global structural patterns that are similar to many real-world networks^4,18,23,37–39^. Furthermore, we demonstrated that while this economy appears to be highly volatile from various traditional perspectives, it actually demonstrates stabilizing, equilibrating patterns. Specifically, this was achieved by employing a network theory approach, analyzing two different aspects of the degree distribution—its scale *γ* and its In-Degree and Out-Degree Ratios, *R*_*in*_ and *R*_*out*_. We found that while *γ* demonstrates a stationary dynamics, *R*_*in*_ and *R*_*out*_ present an under-damped oscillatory convergent dynamics.

The modeling of the system’s dynamics as an under-damped harmonic oscillator provides several insights on the macroscopic characteristics of the system’s evolution. Specifically, the oscillator’s interpretable parameters helped us in quantifying and ascertaining the anti-phased oscillations, the damping rate and the equilibrium state of the system. Nevertheless, since the under-damped harmonic oscillator serves as a meta model, it is incapable of explaining the causes underlying the observed behavior. This might be resolved by developing a generative model that would offer a better understanding of the underlying forces and mechanisms behind the observed anti-phased under-damped oscillatory dynamics^40,41^. The authors intend to pursue this direction in a future research.

Notwithstanding, the findings of the present study are substantial from several different aspects. First, they establish that this complex ecosystem has in fact an overall converging network dynamics with stable network scaling and characteristic evolution, despite the massive endogenous and exogenous forces that constantly act upon it. Second, the established oscillatory dynamics alongside the robust underlying structure indicate that this ecosystem functions as a single coherent financial market. Third, the identified structural and dynamical patterns advocate a strong affinity between the Ethereum trading system and traditional financial markets^28,29^. This in turn suggests that the Ethereum financial economy, which is unique in its availability of fine-grained transactional data, can function as a laboratory for comprehending and analyzing complex traditional markets. We hope that our present work can inspire further studies in this field.

## Methods

### Data

In order to preserve anonymity in the Ethereum Blockchain^11^, personal information is omitted from all transactions. A user, represented by their wallet, can participate in the economic activity through an address, which is attained by applying *Keccak-256* hash function^42^ on his public key. The Ethereum Blockchain enables users to send transactions in order to either send Ether to other wallets, create new Smart Contracts^12–14^ or invoke any of their functions. Since Smart Contracts are scripts residing on the Blockchain as well, they are also assigned a unique address. A Smart Contract is called by sending a transaction to its address, which triggers its independent and automatic execution, in a prescribed manner on every node in the network, according to the *data* that was included in the triggering transaction.

Smart Contracts representing the examined traded assets comply with the ERC20 protocol^43^ defining the manner in which the asset is transferred between wallets and the form in which data within the asset is accessed. Among these requirements, is the demand to implement a *transfer* method, which will be used for transferring the relevant asset from one wallet to another. Therefore, each transfer of a asset will be manifested by a wallet sending a transaction to its relevant Smart Contract. The transaction will encompass a call to the *transfer* method in its *data* section, containing the amount being transferred and its recipient wallet. Each such asset transfer results in altering the ’asset’s balance’, which is kept and updated in its corresponding Smart Contract’s storage.

We obtain the assets’ transactions (similarly to our previous works^24,44^) basing on the further requirement of the ERC20 protocol, demanding that each call to the *transfer* method will be followed by sending a *Transfer* event and updating the event’s logs with all relevant information regarding the asset transfer. We therefore call an Ethereum full node’s JSON API and fetch all logs matching to the *Transfer* event structure. Parsing these logs result in the following fields per transaction: *Contract Address* - standing for the address of the Smart Contract defining the transferred asset, *Value* - specifying the amount of the asset being transferred, *Sender* and *Receiver* addresses, being the wallet addresses of the asset’s seller and buyer, correspondingly.

We have retrieved all assets transactions spreading between February 2016 and January 2019, resulting in 179, 488, 619 asset trades, performed by 27, 888, 847 unique wallets, trading 11, 900 *active* asset addresses, where an *active* asset was defined by us as participating in at least 100 trades. The dataset of assets transactions is extremely diverse and wide-ranging, resulting in a total of 79, 451 unique asset addresses taking part during the examined timespan, with extremely varied characteristics. For instance, the assets differ in their age, their economic value, activity volume and number of asset holders, some merely serve as test-runs, others aren’t tradable in exchanges yet, and some, according to popular literature, are frauds, all residing next to actual real-world valuable assets. The restriction of our analysis to *active* assets is aiming to focus our analysis to the economy formed merely by real-valuable assets.

### Graph analysis

In order to perceive the network’s structure and assess the connectivity of its nodes, one should examine the network’s degree distribution, considering both in-degree and out-degree, indicating the number of incoming and outgoing connections, correspondingly. The degree distribution *P*(*k*) signifies the probability that a randomly selected node has precisely the degree *k*.

In random networks of the type studied by Erdös and Rényi^45^, where each edge is present or absent with equal probability, the nodes’ degrees follow a *Poisson* distribution. The degree obtained by most nodes is approximately the average degree $\bar{k}$ of the network. These properties are also manifested in dynamic networks^46^. In contrast to random networks, the nodes’ degrees of social networks (such as the Internet or citation networks) often follow a *power law* distribution^16^:

9 $$P(k)={k}^{-\gamma }$$

The power law degree distribution indicates that there is a non-negligible number of extremely connected nodes even though the majority of nodes have small number of connections. Therefore the degree distribution has a long right tail of values that are far above the average degree. Power law distributions can be found in many real networks, Newman^23^ summarized several of them, including word frequency, citations, telephone calls, web hits, or the wealth of the richest people. In this work however, we found that the vast majority of examined networks are better fitted by a truncated power-law (power-law with cutoff):

10 $$P(k)={k}^{-\gamma }\cdot {e}^{-\beta k}$$

### Truncated power-law fit

We applied a prevalent statistical framework^36^ for fitting the degree distributions to an adequate model. Specifically, we used a maximum-likelihood fitting method for parameters’ estimation and employed goodness-of-fit tests based on likelihood ratios in order to compare competing models and evaluate which better fits our data. The log likelihood ratio test calculates the likelihood of the given data between two competing distributions. The logarithm of this ratio is positive or negative depending on which model presents a better fit, or is zero if a tie is obtained. The sign of the LLR is subject to statistical instability and when close to zero, the fluctuations can change its sign. In order to establish the statistical significance of the LLR sign, we calculate its standard deviation and corresponding p-value, where small p-values( < 0.1) indicate that the established sign is a reliable estimate of model compatibility.

### Oscillation dynamics

We consider the Ethereum financial ecosystem as a social physical system and thus use physical models to analyze it, similarly to^29,37,47,48^. We hypothesize that it behaves as a dynamical system approaching its equilibrium state, which can be modeled as a damped harmonic oscillator.

A harmonic oscillator is a system acted upon by a force negatively proportional to its perturbation from its equilibrium state. Physical systems that are modeled in this way are springs and swings. Systems that also experience a velocity-dependent friction-like force, e.g. air resistance, are modeled by a damped harmonic oscillator. The dynamical equation for these models is:

11 $$m\frac{{d}^{2}x}{d{t}^{2}}=-kx-c\frac{dx}{dt}$$

where *x* is the perturbation from equilibrium, *m* is the mass, *k* is the spring constant and *c* is the viscous damping coefficient. The resonant frequency of the system is defined as ${\omega }_{0}=\sqrt{k/m}$ and represents the oscillation of an *u*ndamped system. One can define the damping ratio as $\zeta =\frac{c}{2\sqrt{mk}}$ which represents how strong the damping is, compared to the resonant frequency, such that an over-damped system *ζ* > 1 does not oscillate, but exponentially converges to the equilibrium state, whereas an under-damped system *ζ* < 1 oscillates with a modified frequency ${\omega }_{1}={\omega }_{0}\sqrt{1-{\zeta }^{2}}$ during its exponential convergence. The case of critically damped system *ζ* = 1 is an important one in physics, but does not relate to the analysis presented below.

Given an under-damped oscillator, the dynamics of the system can be described by the following function:

12 $$x(t)=A\cdot {e}^{-{\omega }_{0}\zeta t}\cdot \sin ({\omega }_{0}\sqrt{1-{\zeta }^{2}}t+\varphi )+{x}_{\infty }$$

Here *φ* is the phase of the oscillation and *x*_*∞*_ is the equilibrium state. In this paper, we will use the under-damped oscillator in order to model the dynamics of the Ethereum network meta-parameters *R*_*out*_ and *R*_*in*_ and extract the parameters of its dynamics.

## Supplementary information

Supplementary Information.
