# Supplementary material for: Network Dynamics of a Financial Ecosystem
Source: Sci Rep. 2020 Mar 12;10:4587. doi: 10.1038/s41598-020-61346-y (PMC7067835; doi:10.1038/s41598-020-61346-y)
Supplement: Supplementary file 1 — Supplementary Information. [file 41598_2020_61346_MOESM1_ESM.pdf]

Network Dynamics of a Financial Ecosystem

Shahar Somin, Yaniv Altshuler, Goren Gordon,
Alex 'Sandy' Pentland and Erez Shmueli

February 20, 2020

Supplementary Information

As an initial analysis of the Ethereum economy's network parameters, we examine the basic aspects of edges and nodes in the network. Fig S1 represents both static and dynamic views on these aspects. Panel A presents the dynamics of the number of nodes and edges of weekly transactions graphs along the entire examined time. Panels B and C depict the distribution of nodes and the distribution of edges among all networks, respectively. Panel D presents the joint distribution of edges and nodes across networks, indicating the two phases evident in the economy size, occurring around April 2017.

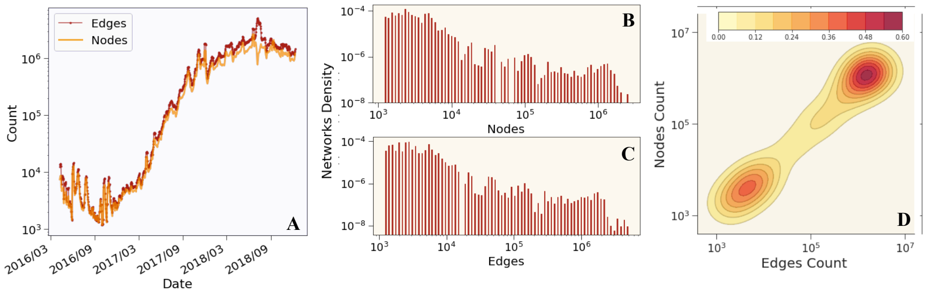

Figure S1: Analysis of amounts of edges and nodes in weekly transactions graphs. Panel A depicts the dynamics of nodes and edges amounts along the examined 2.5 years. Panels B and C depict the distribution of nodes and the distribution of edges across weekly networks, respectively. Panel D presents the joint distribution of edges and nodes across weekly networks.

We further analyze another network parameter capturing the ratio between max-connected and 1-connected traders, referred to as In-Degree Ratio and Out-Degree Ratio. We model their dynamics along time using an under-damped harmonic oscillator. The under-damped harmonic oscillator model is governed by five parameters: (i) $\lambda = \omega_0\zeta$ representing the exponential decay, (ii) $\omega = \omega_0\sqrt{1-\zeta^2}$ standing for the angular frequency, (iii) R_∞ for the stable state to which the system converges, (iv) A representing the maximal amplitude of the

oscillation and (v) φ for the phase shift. The parameters of the fitted oscillators to R_{in} and R_{out} are presented in Table S1.

Table S1: Under-Damped Oscillator Models' Parameters

Parameter	osc_{in}	osc_{out}
A	0.40	-0.75
φ	2.74	2.36
R_{∞}	1.38	1.08
$\frac{2\pi}{\omega_0}$ (days)	365.3	344.3
ζ	0.14	0.26
$\frac{1}{\lambda}$ (days)	402.2	204.6
$\frac{2\pi}{\omega}$ (days)	369.2	357.4
k	2.96e−4	3.33e−4
c	4.97e−3	9.77e−3

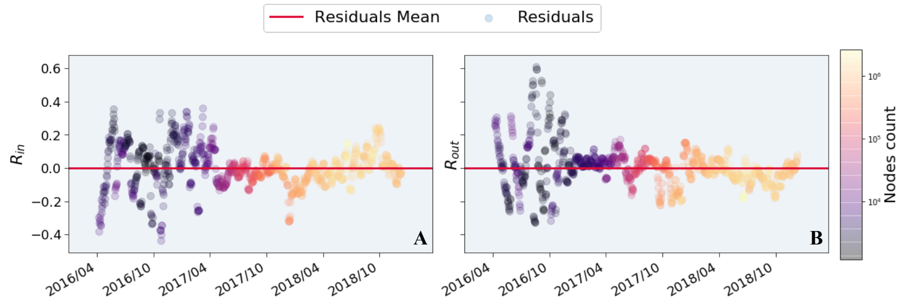

Figure S2: Residuals plots for the Oscillator model's fit to R_{in} and R_{out} along time t are presented in panels A and B, correspondingly, with coloring depending on network size. Both depict a symmetric dispersion around the 0 mean.

In order to examine how well the Oscillator model describes the dynamics of In-Degree and Out-Degree Ratios along time, we explore the residuals from the fit, i.e the deviations of the dependent variable, $osc(t)$, from the fitted oscillator for each day, t:

$$Residual(osc(t)) = R(t) - osc(t) \tag{1}$$

Fig. S2 displays the residuals from the under-damped oscillator fit, for both R_{out} and R_{in}. Indeed both fits present symmetric dispersion of values around an

approximately zero mean throughout the entire time and a decreasing variance of noise along time. This validates our choice of the under-damped harmonic oscillator for modeling the In-Degree and Out-Degree Ratios.

We aim to verify the extent to which the network's size influences the damping oscillatory dynamics undergone by the network. For that intent, we analyze R as a function of network size, N, as is presented in panels (C) and (D) of Fig. S3, for R_{in} and R_{out} correspondingly. Both present a 2-phased behaviour of R as a function of N, separated by a threshold at $N_0 = 10^4$. The first phase manifests a random dispersion of R values, along different N-s, while the second phase consists of quite constant trend in R values.

We postulate that the random phase of R indicates that the oscillations observed in R along time, are not N dependent. This assumption can be validated by observing panels (A) and (B) in Fig. S3, presenting the oscillating dynamics of R_{in} and R_{out} along time, with color-map indicating the size of the corresponding network G_t. Evident from this analysis is that most of the network's oscillating nature occurs prior to the time when network size reaches the $N_0 = 10^4$ threshold. We conclude that though network size seems to have an influence on the oscillator's damping rate, evident from the stable phase presented in panels (C) and (D), it does not act as the source of the oscillating nature of the network's dynamics.

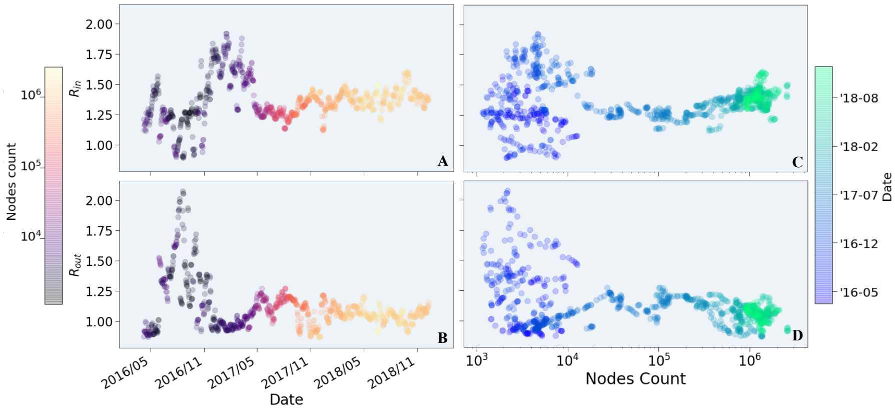

Figure S3: Network's size influence on R dynamics, validating the hypothesis that N does not govern R's oscillating nature, though it probably influences its damping rate. Panels A and B depict R_{in} and R_{out} dynamics along time, with coloring depending on corresponding network size. Panels C and D depict R_{in} and R_{out} as a function of network size, correspondingly, presenting 2 phased dynamics. First phase presents a random dispersion of R along different N values, while second phase depicts a rather constant trend of R.

We further wish to investigate the underlying mechanism behind the different characteristics of R_{in} and R_{out}, including their anti-phased dynamics

and the over-damped values of R_{out}. We start by addressing the semantic economic interpretation of the aforementioned ratios. Specifically, the max in-connected node CN_{in}^{max} represents the maximal buying hub, and correspondingly for CN_{out}^{max} and maximal selling hub. Similarly, CN_{in}^{1} and CN_{out}^{1} represent lightly-connected buyers and lightly-connected sellers, respectively. Panel A in Fig. S4 depicts the high correlation between $deg(CN_{in}^{max})$ and $\#CN_{out}^{1}$, i.e between max buying hub and lightly-connected sellers. Similarly, panel B in Fig. S4 presents the correlation between $deg(CN_{out}^{max})$ and $\#CN_{in}^{1}$, i.e between max selling hub and lightly-connected buyers. These observations manifest the inherent connection between buyers and sellers in the network and might offer an explanation to their anti-phased dynamics. For example, a low R_{out} might result from a large selling hub, which is accompanied with a large number of lightly-connected buyers, resulting in a high R_{in}.

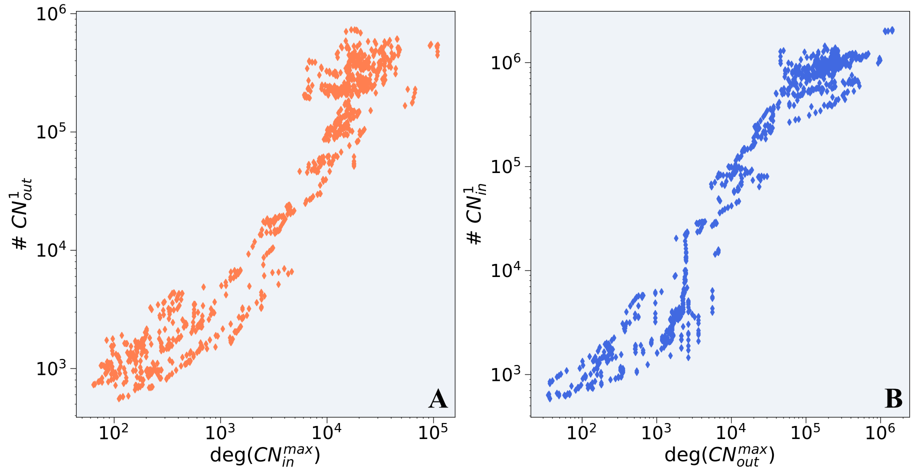

Figure S4: Buyers and sellers connections. Panel A depicts the dependency between the degree of the max-in-connected node and the number of 1-out-connected nodes. Panel B presents the dependency between the degree of the max-out-connected node and the number of 1-in-connected nodes. Both panels manifest high correlations between the examined quantities.

We further note that Fig. S5 might help account for the strong damping effect evident in R_{out} dynamics, as opposed to the slower damping effect apparent in R_{in} dynamics. Panel B depicts the dynamics of the max-in-connected node's degree and the number of 1-in-connected nodes along time. In particular, it reveals that as of April 2017 extremely large max-in-connected nodes started emerging and were accompanied by a similar rise in the number of 1-in-connected nodes. This leads to a low and approximately constant in-degree ratio, prompting the damping of oscillations and the lowering of the convergence value of R_{in}.

Similarly, panel A describes the dynamics of the max-out-connected node's degree and the number of 1-out-connected nodes along time. Specifically it manifests that these two quantities stabilize, with $\#CN_{in}^{1}$ being much higher than $deg(CN_{in}^{max})$, only as of November 2017. This in turn inflicts on R_{in}, thereby proposing a possible explanation to the much slower damping of R_{in} oscillations and to $R_{in}^{\infty} > R_{out}^{\infty}$. We conclude that the oscillatory nature of both ratios dynamics is strongly related to the buyers-sellers connection. Specifically, the oscillations, which might have been triggered by the initial shock presented to the system upon its creation, continue until stabilization due to the transition between a *buyers* market to a *sellers* market and vice-versa.

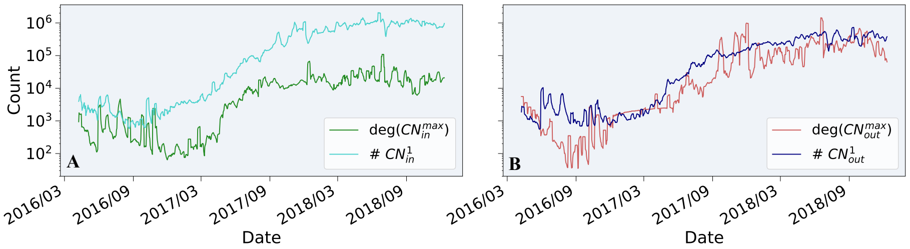

Figure S5: Dynamics of the max-connected node and 1-connected nodes. Panel A presenting the dynamics of max-in-connected node degree and the amount of 1-in-connected nodes. Panel B demonstrates the dynamics of max-out-connected node and the amount of 1-out-connected nodes.
